# Attenuated XPC Expression Is Not Associated with Impaired DNA Repair in Bladder Cancer

**DOI:** 10.1371/journal.pone.0126029

**Published:** 2015-04-30

**Authors:** Kishan A. T. Naipal, Anja Raams, Serena T. Bruens, Inger Brandsma, Nicole S. Verkaik, Nicolaas G. J. Jaspers, Jan H. J. Hoeijmakers, Geert J. L. H. van Leenders, Joris Pothof, Roland Kanaar, Joost Boormans, Dik C. van Gent

**Affiliations:** 1 Department of Genetics, Erasmus University Medical Center, Rotterdam, The Netherlands; 2 Department of Pathology, Erasmus University Medical Center, Rotterdam, The Netherlands; 3 Department of Radiation Oncology, Erasmus University Medical Center, Rotterdam, The Netherlands; 4 Department of Urology, Erasmus University Medical Center, Rotterdam, The Netherlands; 5 Cancer Genomics Netherlands, Erasmus University Medical Center, Rotterdam, The Netherlands; University Medical Center Hamburg-Eppendorf, GERMANY

## Abstract

Bladder cancer has a high incidence with significant morbidity and mortality. Attenuated expression of the DNA damage response protein Xeroderma Pigmentosum complementation group C (XPC) has been described in bladder cancer. XPC plays an essential role as the main initiator and damage-detector in global genome nucleotide excision repair (NER) of UV-induced lesions, bulky DNA adducts and intrastrand crosslinks, such as those made by the chemotherapeutic agent Cisplatin. Hence, XPC protein might be an informative biomarker to guide personalized therapy strategies in a subset of bladder cancer cases. Therefore, we measured the XPC protein expression level and functional NER activity of 36 bladder tumors in a standardized manner. We optimized conditions for dissociation and *in vitro* culture of primary bladder cancer cells and confirmed attenuated XPC expression in approximately 40% of the tumors. However, NER activity was similar to co-cultured wild type cells in all but one of 36 bladder tumors. We conclude, that (i) functional NER deficiency is a relatively rare phenomenon in bladder cancer and (ii) XPC protein levels are not useful as biomarker for NER activity in these tumors.

## Introduction

Bladder cancer (BC) is the fifth most common malignancy in Europe with an incidence of more than 150,000 cases resulting in more than 50,000 deaths per year [1]. Bladder tumors present either as non-muscle-invasive (NMIBC) (70%) or as muscle-invasive bladder cancer (MIBC) (30%) [2]. MIBC is a potentially lethal disease with a 5-year survival rate of 50–60% [3]. Therapy of MIBC involves mainly surgery and systemic chemotherapeutic treatments, the latter mainly for recurrent and metastatic disease.

There is a clear clinical need for the development of new systemic treatment strategies for MIBC, as the survival of MIBC patients has not improved over the past decades. Treatments targeting tumor-specific pathways have shown to be effective in various cancers, such as breast, ovarian and renal cancer but for BC such therapies are not available. Therapies targeting DNA damage response (DDR) mechanisms are promising approaches in anticancer treatment [4]. DDR mechanisms are essential for the maintenance of genomic stability through DNA repair, cell cycle regulation and apoptosis. The functionality of specific DDR pathways is an important parameter that contributes to the sensitivity of malignant cells to DNA damaging chemo- and radiotherapy. In addition, targeting specific defects in DDR pathways might result in a more effective treatment. For example, the DDR defect caused by *BRCA1* or *BRCA2* mutations in breast and ovarian cancer sensitizes these tumors to inhibitors of poly(ADP-ribose)polymerase (PARP) [5,6]. Thus, determining the DDR status of individual tumors can be valuable for the anti-cancer treatment choice.

Nucleotide excision repair (NER) is responsible for the repair of a wide range of different types of DNA damages produced by environmental mutagenic and carcinogenic agents. Structurally unrelated lesions repaired by NER include UV induced helix distortions, bulky DNA adducts and intrastrand crosslinks (e.g. induced by drugs such as Cisplatin) [7]. Two major subpathways of NER include global genome NER (GG-NER) and transcription coupled NER (TC-NER). Xeroderma Pigmentosum complementation group C (XPC) is specifically involved in GG-NER and serves as a damage recognition protein in complex with HR23B. Only after damage recognition by XPC-HR23B the core NER proteins are recruited to the lesion and NER is carried out [8]. This suggests that XPC is the rate limiting factor in GG-NER.

Several lines of evidence suggest that NER genes play a role in the development and progression of BC; polymorphisms in some NER genes increase BC risk, especially in smokers [9–12]. Furthermore, it was shown that attenuated expression of the NER protein XPC is present in ~40% of all bladder tumors and a contributing factor in tumor progression [13]. Also, hypermethylation of the *XPC* gene promoter is significantly higher in BC compared to normal mucosa and is associated with higher pathological stage, presence of metastasis and p53 mutations [14]. These observations suggest that specific *XPC* defects in BC cause defective NER and a poor outcome in a subgroup of tumors. On the other hand, *XPC* defects might be exploited for individualized targeted chemotherapy. By selectively targeting the NER defect using compounds that induce specific DNA damage whose repair requires NER, e.g. Cisplatin, increased cytotoxicity could be achieved in the NER deficient tumor cells.

In the present study, we aimed to identify XPC expression levels in individual clinical BC samples along with functional NER activity, as a starting point for individualized therapy. We established a reproducible method to obtain a short-term single layer cell culture from fresh bladder tumor specimens to perform our assays and conclude that low XPC expression not necessarily results in defective NER.

## Materials & Methods

### Collection of human bladder cancer samples

BC samples (n = 105) were collected at Erasmus MC Rotterdam. Nine tumor samples were obtained by radical cystectomy and the remaining by Trans Urethral Resection (TUR). Upon harvesting, the tumor specimens were directly transported to the laboratory in transport medium (RPMI-1640, 10% fetal calf serum and antibiotics). All samples were then given a unique code and the researchers were blinded for the patient data. All tumor samples were evaluated after standard HE staining and tumor stage and grade were assessed by the pathologist according to the WHO classification 1973 and 2009.

Bladder specimens were collected as surgical residual material. According to hospital policies patients were made aware that residual material can be used for research purposes unless patients choose not to participate. All materials were coded in such a way that researchers could not trace back information to the individual patient. Therefore, no explicit written/oral informed consent was obtained and the need for written/oral informed consent was waived by the Institutional Review Board of Erasmus MC and this specific research project and procedure was approved by the IRB (METC-2012-113).

### Tissue culture

Tumor specimens were manually sliced into smaller pieces using surgical scissors and subsequently subjected to dissociation using Miltenyi Biotec Tumor Dissociation Kit in combination with a gentleMACS Dissociator according to manufacturer’s protocol. Cell suspensions were seeded on glass cover slips in AmnioMax-C100 medium (Gibco Life Technologies, Carlsbad, CA) and incubated overnight at 37°C, 5%CO_2_ and atmospheric oxygen. If sufficient numbers of tumor cells were attached, a small number of wild-type human fibroblasts, C5RO, were added to the culture to serve as internal controls for standardized unscheduled DNA synthesis (UDS) assay which was performed the next day. To distinguish the C5RO cells from tumor cells, their cytoplasm was beforehand labeled with 2.0μm polystyrene beads [15].

Bladder cell lines were obtained from ATCC (HT-1197 [ATCC CRL-1473] and T24 [ATCC HTB-4] and cultured in RPMI1640 medium supplemented with 10% FCS and antibiotics.

### Unscheduled DNA Synthesis (UDS) assay

UDS assay was performed by UV-radiating cells with 16 J/m^2^ UVC light and subsequently labeling cells for three hours with 20μM 5-ethynyl-2'-deoxyuridine (EdU, a thymidine analogue) in the culture medium (Hams F10 without thymidine, 10% dialyzed FCS, antibiotics) [16,17]. Afterwards, cells were washed and incubated in medium supplemented with 10μM thymidine for 15 minutes to eliminate non-specific EdU binding. Cells were fixed using 3,7% formaldehyde in PBS containing 0.5% Triton X-100. EdU incorporation was visualized by fluorescence microscopy using Click-it chemistry (Life Technologies) according to the manufacturer’s protocol and quantified by image analysis using FIJI (ImageJ).

### Immunofluorescence staining

To visualize XPC-protein, fixed cells were incubated with a primary antibody against XPC (Santa Cruz D-10; sc-74410 diluted 1/1000) for 90 min and a secondary Alexa 488 or 594 conjugated antibody for 60 min, prior to mounting in DAPI containing mounting medium (Vectashield). Specificity of the antibody was tested on XPC wild type (C5RO) vs XPC deficient (XP21RO) cells.

### Image analysis

XPC as well as UDS staining of at least 50 individual tumor cell nuclei was quantified by standard imaging software (ImageJ) and compared with at least 20 C5RO nuclei on the same slide. Subsequently, standardized XPC and UDS levels of tumor cells were calculated as:

X=(avg. intensity tumor cells/ avg. intensity C5RO cells) X 100%.

### Immunohistochemistry staining

To visualize XPC on formalin-fixed paraffin embedded (FFPE) sections of bladder tumors, sections were heated to 95°C for 15 min with antigen retrieval buffer (DAKO S1699) and permeabilized with 0.5% Triton X-100 in PBS for 20 minutes. Primary antibody (anti-XPC Santa Cruz D-10; sc-74410 diluted 1/1000) was added, followed by incubation overnight at 4°C. Secondary HRP antibodies were incubated for 60 min at room temperature and visualized using DAB peroxidase chemistry (DAKO K6438). The samples were quantitatively analyzed using an immunohistochemical scoring system (S1 Fig). This immunoreactivity scoring (IRS) system takes into account the percentage of positive cells and the intensity of the observed staining [18].

### Immunoblotting

Immunoblotting was performed using polyclonal rabbit anti-XPC [19] and mouse monoclonal anti-Tubulin (clone: B-512, Sigma-Aldrich) for one hour at room temperature, followed by incubation with secondary antibodies IRDye 800CW Donkey anti-Mouse IgG (H+L) (LI-COR) and IRDye 680RD Donkey anti-Rabbit IgG (H+L) (LI-COR) for one hour at room temperature in the dark. Immunoreactive bands were visualized using Odyssey 3.0 (LI-COR).

### Colony survival

150 cells per well were seeded into six-wells plates and every condition was plated in triplicate. Next day, when cells were attached, they were treated with 0, 2, 4, 6, and 8 J/m^2^ UVC. After 8 days of incubation, the cells were stained with 0.1% Coomassie Brilliant Blue for at least 30 min. Colonies were counted with GelCount (Oxford Optronix).

### Statistical analysis

Statistical analysis was performed using IBM SPSS Statistics v21 and GraphPad Prism v5.

## Results

### XPC expression in bladder cancer

**Fig 1:**
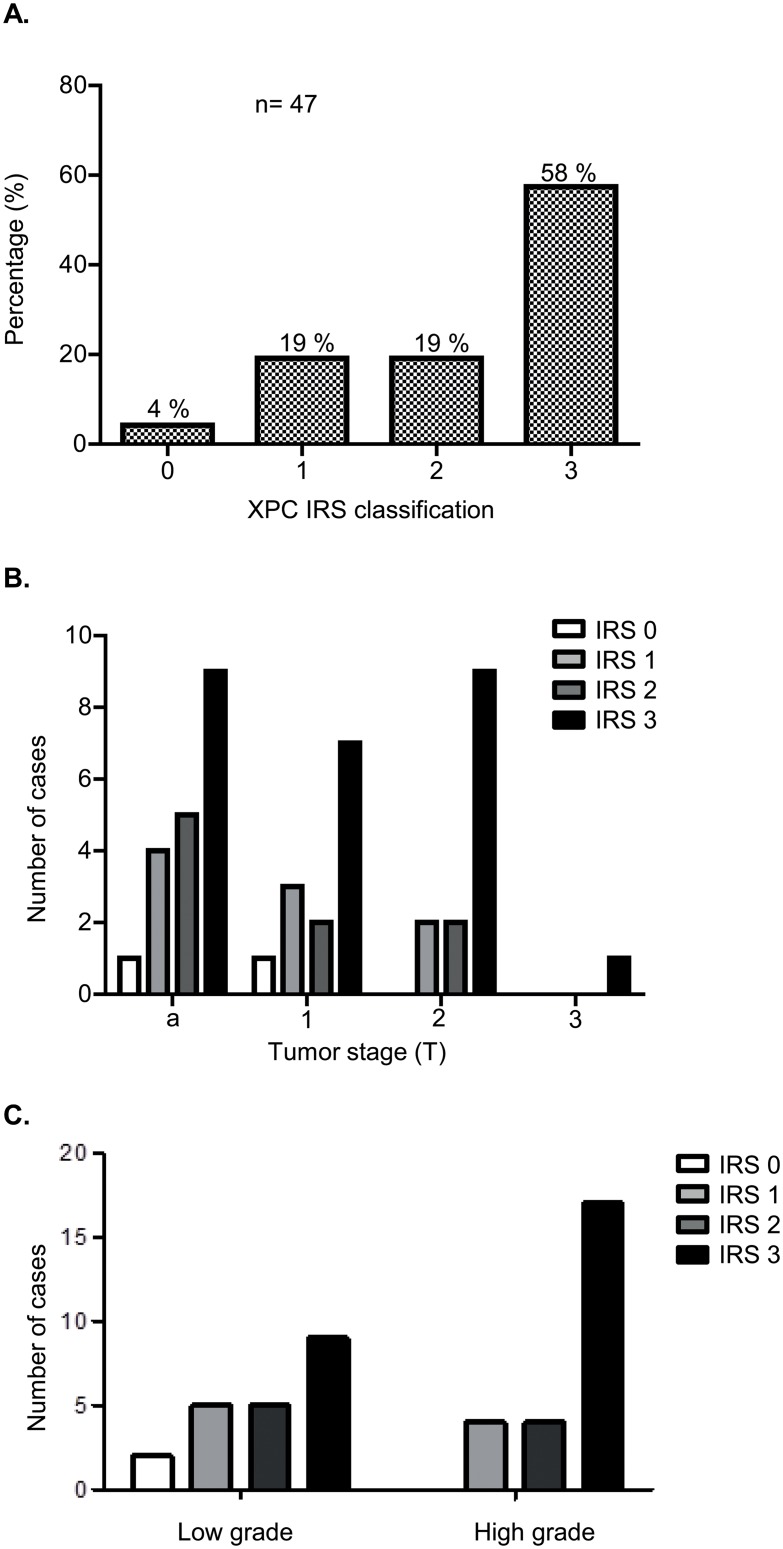
XPC expression levels in human bladder cancer. **A**. Frequency distribution of XPC IRS classifications in 47 TURB samples. **B**. Frequency distribution of XPC IRS classifications based on tumor stage. Differences in IRS classifications were not statistically significant (Kruskal—Wallis test, p = 0.5266). C. Frequency distribution of XPC IRS classifications based on tumor grade. Differences in IRS classifications were not statistically significant (Mann- Whitney test, p = 0.6612).

Forty seven out of 105 collected tumor samples contained sufficient tumor to generate FFPE sections. XPC protein expression was investigated in these 47 tumors by immunohistochemistry and a wide range of XPC levels was observed. The majority (58%) of the samples displayed strong XPC expression (IRS = 3) whereas 38% of the samples displayed lowered levels (IRS = 1 or 2). In 4% (2 cases) only background levels were detected (IRS = 0) (Fig 1A and S1 Fig). XPC levels did not show significant correlation with tumor stage (Kruskal-Wallis; p = 0.53) nor with tumor grade (Mann-Whitney; p = 0.66) (Fig 1B and 1C).

### Optimizing ex vivo culture of dissociated bladder cancer cells

Subsequently, we investigated whether the differences in XPC protein expression correlated with variations in DNA repair capacity. NER activity can be quantified by measuring repair-associated DNA synthesis (also called unscheduled DNA synthesis (UDS)). The UDS assay measures the incorporation of a labeled nucleoside, 5-ethynyl-2'-deoxyuridine (EdU), after exposure to UV-C radiation (predominantly 254nm), and is determined in cells that are not undergoing S-phase-dependent DNA synthesis [20]. The UDS assay requires a single cell layer of cells, because UV-C radiation does not penetrate in thick tissue biopsies. Therefore, a reproducible method was established to obtain a short-term single layer cell culture from fresh bladder tumor specimens.

First we compared several dissociation methods to obtain cell cultures from BC biopsies on glass cover slips. Enzymatic dissociations using various proteases resulted in low success rates with regard to tumor cell attachment. We considered the attachment successful when tumor cells attached to more than 5% of the culture dish surface. Attachment between 5–30% was considered intermediate, whereas more than 30% indicated appropriate attachment (Table 1). We obtained attached tumor cells in only 35% of tumors upon Collagenase VII treatment, whereas no attachment was achieved with Trypsin or Dyspase treatments (Table 1). Notably, in case of cells attaching to the glass cover slip, small clumps of cells attached more efficiently than single cells (S2A Fig). On the other hand, dissociation assays using the Miltenyi Biotec Tumor dissociation kit and the gentleMACS dissociator resulted in single cell layer cultures in approximately 80% of the tumors (Table 1). To distinguish these urothelial tumor cells from fibroblasts that often contaminate such primary cultures, we performed a staining against cytokeratin 18. In case of appropriate attachment the majority of cells stained positive for cytokeratin 18, indicative of a pure urothelial tumor cell culture (S2B Fig). On the other hand, tumor biopsies that displayed cauterization effects on HE staining, indicative of diathermic tumor removal, did not attach to coverslips possibly due to (tumor) cell killing by the excessive heating. Furthermore, tumor biopsy samples derived from cystectomy specimens attached successfully in only one out of nine samples, indicating that invasive bladder tumors are difficult to culture *ex vivo* in a single cell layer.

**Table 1:** Comparison of different dissociation methods.

Type of dissociation	Number of tumors	Tumors from which cells attached to >30% of surface	Tumors from which cells attached to 5–30% of surface	Tumors from which cells attached to 0–5% of surface
Collagenase VII	31	4 (13%)	7 (23%)	20 (65%)
Trypsin	5	0 (0%)	0 (0%)	5 (100%)
Dispase	6	0 (0%)	0 (0%)	6 (100%)
Dissociation kit and dissociator	42	22 (52%)	11 (26%)	9 (21%)

Note: FFPE samples that contained no tumor cells on HE staining are excluded from this table

Different coating strategies of cover slips, such as gelatin, fibronectin or Poly-L-lysine did not lead to better attachment of cells (data not shown). Finally, we tested different cell culture media to increase the attachment of tumor cells. Media composed of DMEM or RPMI-1640 (both supplemented with 10% FCS and antibiotics) showed attachment of cells in less than 50% of samples, whereas AmnioMax-C100 medium achieved attachment in more than 80% of samples (Table 2). The combination of the Miltenyi Biotec dissociation method and AmnioMax-C100 medium was used in all subsequent experiments and resulted in a short-term single layered BC tumor cell culture within two days after tumor removal.

**Table 2:** Comparison of different culture media for tumor cell attachment.

	DMEM	RPMI-1640	AmnioMax
attached	0 (0%)	7 (47%)	32 (84%)
not attached	13 (100%)	8 (53%)	6 (16%)
Total	13	15	38

### UDS in bladder cancer samples

**Fig 2:**
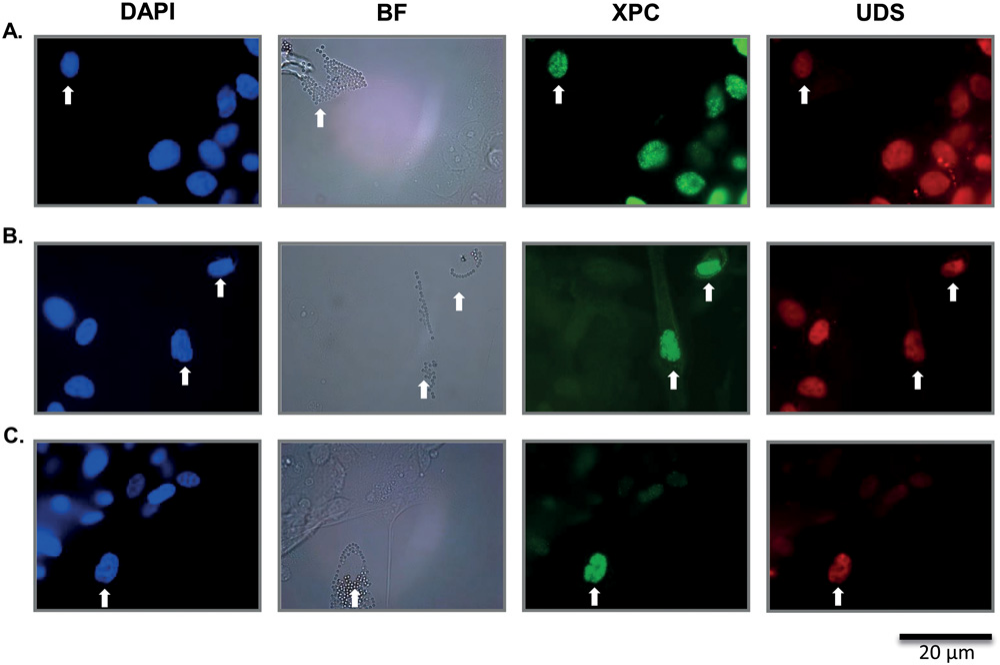
UDS and XPC in bladder cancer samples compared to C5RO. **A**. Upper left image displays all DAPI nuclei. To the right is a bright field (BF) image in which the C5RO cell is labelled with cytoplasmic polystyrene beads. Next: XPC fluorescent staining displaying XPC protein expression of tumor cells to be similar compared to adjacent wild type fibroblast. Upper right image: EdU fluorescent staining displaying the UDS signal of tumor cells to be similar compared to adjacent wild type fibroblast. **B**. Middle row of images represent a bladder tumor sample having lower XPC expression compared to C5RO cells however, normal levels of UDS. **C**. Lower row of images represent a bladder tumor having low XPC expression as well as low UDS levels. White arrow indicates wild type C5RO cell. Other nuclei represent specific bladder cancer sample.

**Fig 3:**
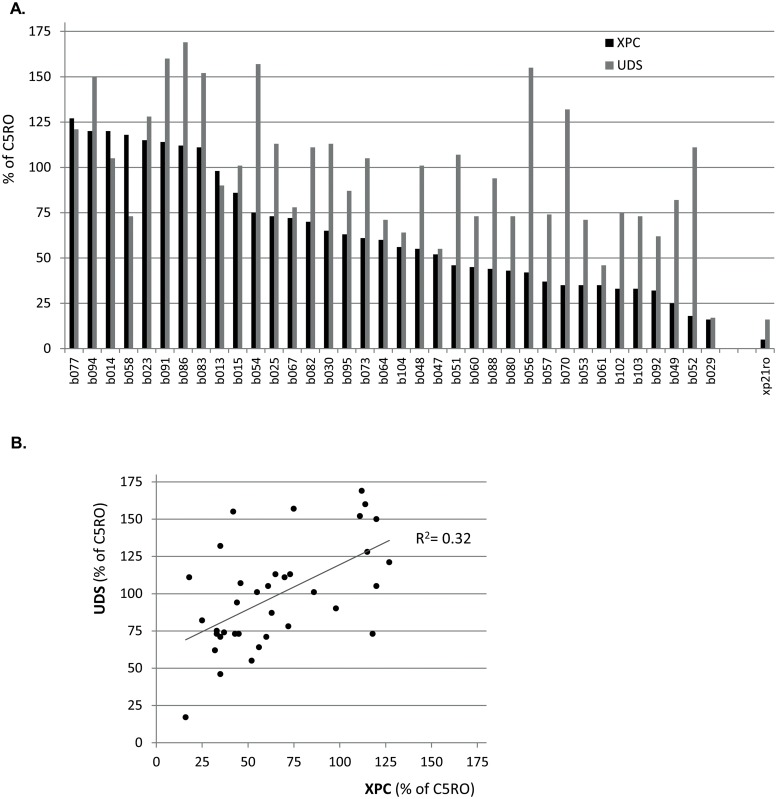
Low XPC expression levels do not correlate with low UDS levels. **A**. Each tumor is represented with its respective score for XPC and UDS levels as a percentage of co-cultured C5RO cells. No tumors displayed XPC levels as low as XP21RO, an XPC deficient cell line. One tumor showed UDS scores similar to XP21RO. **B**. Scatterplot of UDS and XPC scores of each tumor. XPC levels are frequently lower than 50%. Two tumors display UDS levels of lower than 50%, however from one of these UDS levels are close to 50%. The coefficient of determination (R^2^ = 0,32) indicates no or very weak correlation.

Functional NER-activity was analyzed in attached tumor cells from 36 different bladder tumors using the UDS assay. Additionally, we assessed XPC-protein levels in the same cells by immunofluorescent staining. Scoring for XPC and UDS was performed using a scoring method that compared the average staining intensity of tumor cells to that of normal human C5RO fibroblasts, which were co-cultured on the same cover slip as the tumor cells. The fibroblasts in the mixed cultures were labeled with 2 μm polystyrene beads to discriminate them from the tumor cells (Fig 2). Nearly 42% (15/36) of the bladder tumors expressed at least two-fold lower levels of XPC protein than co-cultured fibroblasts (0–50%) (Table 3). Most tumors displayed near normal levels of XPC protein (50–180% of co-cultured fibroblasts). We did not observe XPC staining intensities of less than 5%, a level typical for XPC-deficient fibroblasts (XP21RO) obtained from an XP-patient (Fig 3A). Remarkably, XPC expression showed very weak correlation with UDS in these tumors: R^2^ = 0.32 (Fig 3B). Most tumors with relatively low XPC levels displayed normal UDS levels (Figs 2B and 3). Normal UDS (more than 50% of co-cultured fibroblasts) was observed in 34 out of 36 tumor samples (Table 3). Two tumors showed reduced UDS (less than 50% of co-cultured fibroblast controls), however in one of these the UDS was close to 50%, much higher than observed in XPC^-/-^ cells (Figs 2C and 3A). The other tumor showed XPC and UDS levels similar to that of XPC^-/-^ cells. Unfortunately, due to lack of material no further genetic analysis on this tumor could be performed. Concluding, relatively low levels of XPC expression can still support NER activity and decreased XPC expression does not necessarily influence NER activity in ex vivo cultured BC cells.

**Table 3:** XPC expression levels compared to UDS levels in bladder cancer.

	XPC normal^a^	XPC ↓^b^	Total
**UDS normal** ^a^	21 (58%)	13 (36%)	34 (94%)
**UDS ↓** ^b^	0 (0%)	2 (6%)	2 (6%)
**Total**	21 (58%)	15 (42%)	36 (100%)

^a^ normal = staining intensity is similar to C5RO cells (50%-180%)

^b^ **↓** = staining intensity is less than 50% of C5RO cells

This finding is unexpected in light of a previous publication showing that XPC expression levels varied among a small panel of individual established BC cell lines and that one cell line with low XPC protein level had decreased DNA repair capacity. We obtained this cell line (HT-1197) from the ATCC collection and compared it with a BC cell line with normal XPC expression (T24). Surprisingly, we did not observe a difference in XPC protein levels nor UDS levels among normal fibroblasts, T24 and HT-1197 cells, even though fibroblasts derived from an XP patient (XP21RO) showed clearly reduced XPC protein levels and reduced UDS (S3A Fig). Subsequently, we compared the XPC level of HT-1197 and other known XPC-proficient and -deficient cells by immunoblotting (S3B Fig). Again, HT-1197 did not display a reduced XPC level, whereas the known XPC-deficient cells showed a clear absence of XPC protein. We also analyzed the sensitivity of HT-1197 and T24 to UV-C radiation by performing a colony survival assay and found no difference in sensitivity (S3C Fig). Therefore, we conclude that HT-1197 does not have a decreased XPC protein level nor reduced NER capacity.

### XPC expression and recurrence risk of BC

Finally, to investigate whether tumor recurrence has any impact on XPC protein expression levels we collected additional clinically relevant data and compared XPC protein levels from primary NMIBC to that of recurrent NMIBC. XPC levels did not differ significantly (Mann-Whitney; p = 0.58) between these two groups (S4A Fig). Also XPC protein levels did not differ significantly (Mann-Whitney; p = 0.77) between tumors that showed recurrence within one year and tumors that did not. These data were generated from patient follow-up, regardless of whether an analyzed BC sample was a primary tumor or a recurrence (S4B Fig). This implies that XPC expression levels poorly correlate with the risk of recurrence of NMIBC.

## Discussion

In line with previous studies [13,14], we found that attenuated XPC protein expression is a common phenomenon in BC, both in dissociated tumor cells as well as in FFPE samples. However, previous studies did not analyze functional NER activity in BC. Therefore, we developed an *ex vivo* cell culture system to perform UDS assays, a functional test for NER activity. Here we confirm that XPC expression varied widely among primary BC samples but surprisingly, we found that lowered XPC protein levels did not generally influence NER capacity in BC. More importantly, functional NER deficiency appears to be much less frequent in BC than would be expected based on earlier studies. Reduced levels of XPC protein in these tumors seemed sufficient to functionally support NER. In addition, we did not observe a correlation between XPC expression and tumor grade or stage, nor did we observe a relation with tumor recurrence risk. We conclude that XPC protein levels should not be used as a biomarker to select bladder tumors for impaired DNA repair capacity.

In the present study, we show that severe functional NER defects that confer hypersensitivity to chemicals causing NER-repairable damage, are rare in BC. Therefore, it is unlikely that impaired NER activity can be exploited therapeutically in BC. Furthermore, the frequency of XPC defects in BC may be overestimated in literature, as we did not find an XPC nor NER defect in the HT-1197 cell line, of which previously was reported to harbor such defects [13]. Differences in detection method of XPC expression levels or DNA repair capacity might explain these discrepancies. Nevertheless, many reports suggest that *XPC* gene polymorphisms can influence BC risk or prognosis [10,12,21]. It is still unknown whether these polymorphisms influence XPC expression but it might be that the lowered XPC expression and polymorphisms have a subtle influence on NER activity, which could contribute to increased mutagenesis and carcinogenesis, especially in cases of exposure to certain DNA damaging agents e.g. cigarette smoke. Our study is the first one to describe the correlation between functional NER activity and XPC protein levels measured directly in primary bladder cancer. In our assay, NER activity of more than 50% (of co-cultured fibroblasts) was considered normal and a mild decrease will not be picked up. On the other hand, UDS analyses performed on several NER gene defects, in mouse as well as human cells, show significantly lowered NER activities (less than 50% of co-cultured fibroblasts). This concludes that the UDS assay is suitable to detect a NER deficiency. In the XP21Ro cells apparent XPC levels of around 5% correlated well with the 10% residual NER activity. None of the tumors analyzed showed this low level of XPC, suggesting that the observed XPC levels are able to support near normal NER and that XPC is probably not rate limiting in these tumors. The ex vivo primary tumor culture model used in this study was especially designed and optimized in order to accurately analyze the NER characteristics of individual bladder tumor samples. The primary tumor was dissociated and the cells were cultured on glass cover slips to facilitate standardized UDS assays and XPC measurements by immunofluorescent imaging. This culture system showed robust reproducibility and therefore can be used to screen clinical tumor specimens using functional *ex vivo* assays. The high success rate of this method prevents introduction of a selection bias caused by outgrowth of only a subgroup of clinical cancer specimens. Therefore, this culture method can contribute to the identification of biomarkers that can predict response to anti-cancer treatments

Our experimental design has the advantage that other cells can be co-cultured with the tumor cells as internal controls, eliminating experimental variability between samples. In fact, multiple control cells can be incorporated using polystyrene beads of different size or fluorescent color. In this way we also noticed that one tumor with lower UDS showed higher levels of residual repair capacity than an XPC^-/-^ cell, indicating that NER was not completely abrogated in that sample.

Thus, the important first conclusion from our results is that XPC levels cannot be used as a biomarker to guide therapy choice or treatment response to NER-repairable DNA-damaging agents in BC. We also conclude that the *ex vivo* culture system and UDS assay are well-suited tools to identify tumors (other than BC) with impaired NER activity. Furthermore, novel functional DNA repair assays could be developed to assess the activity of other DNA repair pathways in tumor cells and ultimately identify DNA repair defects that could be exploited therapeutically. Notably, this study underlines the major importance of functional assays to evaluate the results of more indirect measurements, such as RNA and protein expression levels.

## Supporting Information

S1 Fig Immunoreactivity scoring system (IRS). **A**. IRS scoring system. **B**. Representative pictures of XPC staining on bladder cancer samples for different categories of IRS
(PDF)

S2 Fig Small clumps of cells attaching to cover slips. **A**. Representative bright field images showing different clumps of the same tumor attaching to the cover slip and spreading out. **B**. In case of appropriate attachment the majority of cells stained positive for cytokeratin 18, indicating a pure tumor cell culture and no contaminating fibroblasts. Blue = DAPI, Green = cytokeratin 18.
(PDF)

S3 Fig HT-1197 displays normal XPC protein and UDS levels. **A**. Immunofluorescent images comparing XPC and UDS levels of XP21RO, T24 and HT-1197 cells to that of C5RO cells. XP21RO are known to be deficient in XPC and UDS. No difference is seen between T24 and HT-1197. Blue = DAPI, BF = Bright Field image showing C5RO cells labeled with 2 μm polystyrene beads, Green = XPC expression level, Red = UDS level. White arrows indicate the nuclei of the respective cell line. **B**. Immunoblot for XPC comparing HT-1197 to T24, known XPC deficient and wild type fibroblasts. **C**. Colony survival assay showing colony formation capacity of HT-1197 and T24 cells after UV radiation. No difference between HT-1197 and T24 is observed. Error bars indicate the standard deviation based on four independent experiments.
(PDF)

S4 Fig XPC protein levels and recurrence of tumors. **A**. Tumors were split in two groups, primary tumors and recurrent tumors, and standardized XPC protein expression scores were compared from these groups. No significant difference was observed between these groups. Mann-Whitney test, p = 0.58. **B**. Patient follow up data indicating no difference between XPC levels from tumors that would not recur within one year of TUR and tumors that would recur. Each data point represents one tumor. Horizontal line represents median and error bars indicate interquartile range.
(PDF)
