# Supplementary material for: Attenuated XPC Expression Is Not Associated with Impaired DNA Repair in Bladder Cancer
Source: PLoS One. 2015 Apr 30;10(4):e0126029. doi: 10.1371/journal.pone.0126029 (PMC4416023; doi:10.1371/journal.pone.0126029)
Supplement: S1 Fig — A. IRS scoring system. B. Representative pictures of XPC staining on bladder cancer samples for different categories of IRS (PDF) [file pone.0126029.s001.pdf]

A.

Percentage of positive cells	*X Intensity of Staining*	*= Score (0 – 12)*	*IRS – classification*
0 = no positive cells	0 = no color reaction	0 – 1 = negative	0 = negative
1 = < 10% of positive cells	1 = mild reaction	2 – 3 = mild	1 = positive, weak expression
2 = 10-50% positive cells	2 = moderate reaction	4 – 8 = moderate	2 = positive, intermediate expression
3 = 51-80% positive cells	3 = intense reaction	9 – 12 = strongly positive	3 = positive, strong expression
4 = > 80% positive cells			

B.

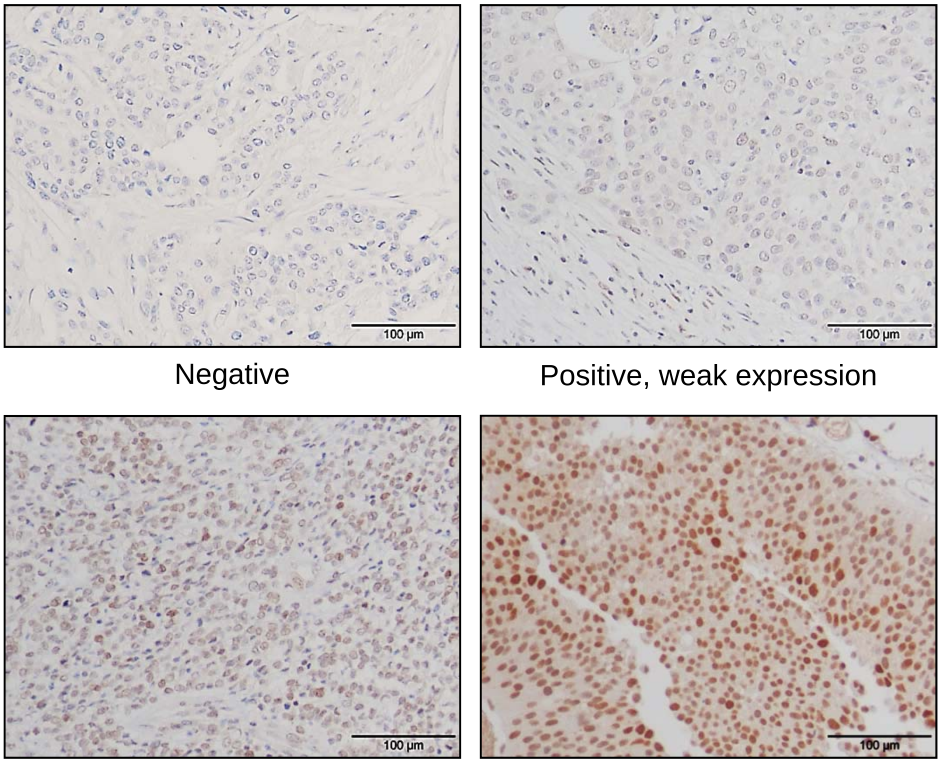

Negative | Positive, weak expression

Positive, intermediate expression | Positive, strong expression

Figure S1: Immunoreactivity scoring system (IRS)
A. IRS scoring system. **B.** Representative pictures of XPC staining on bladder cancer samples for different categories of IRS
