# Supplementary material for: Attenuated XPC Expression Is Not Associated with Impaired DNA Repair in Bladder Cancer
Source: PLoS One. 2015 Apr 30;10(4):e0126029. doi: 10.1371/journal.pone.0126029 (PMC4416023; doi:10.1371/journal.pone.0126029)
Supplement: S2 Fig — A. Representative bright field images showing different clumps of the same tumor attaching to the cover slip and spreading out. B. In case of appropriate attachment the majority of cells stained positive for cytokeratin 18, indicating a pure tumor cell culture and no contaminating fibroblasts. Blue = DAPI, Green = cytokeratin 18. (PDF) [file pone.0126029.s002.pdf]

A.

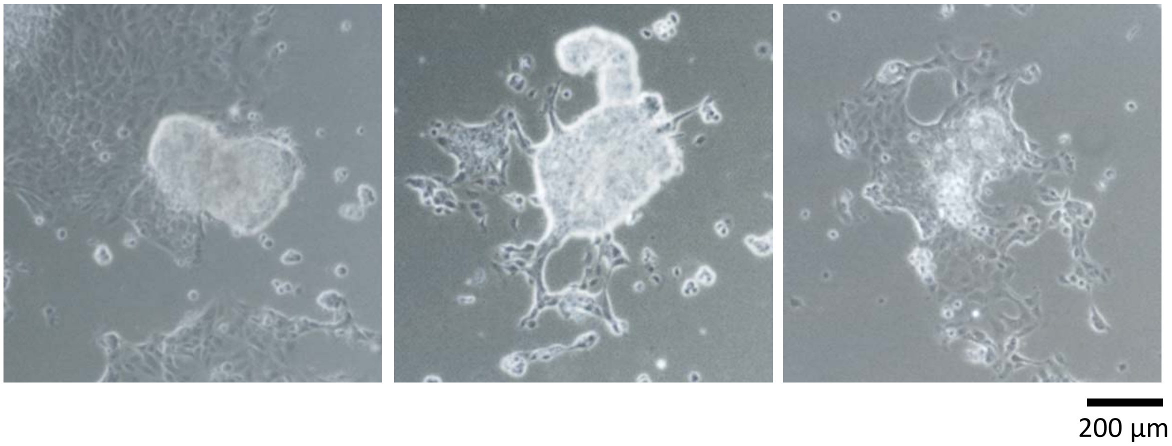

B.

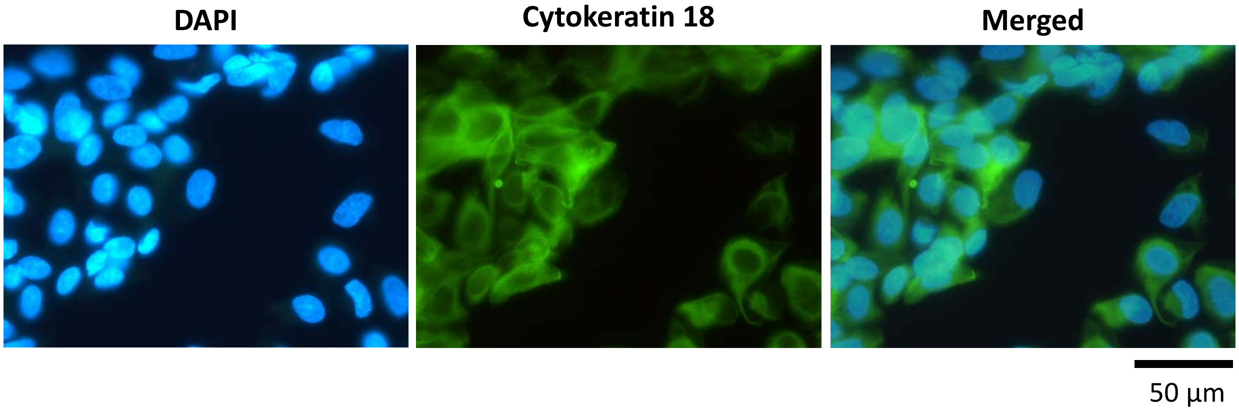

Figure S2: Small clumps of cells attaching to cover slips
A. Representative bright field images showing different clumps of the same tumor attaching to the cover slip and spreading out. **B**. In case of appropriate attachment the majority of cells stained positive for cytokeratin 18, indicating a pure tumor cell culture and no contaminating fibroblasts. Blue= DAPI, Green=cytokeratin 18.
