# Supplementary material for: Attenuated XPC Expression Is Not Associated with Impaired DNA Repair in Bladder Cancer
Source: PLoS One. 2015 Apr 30;10(4):e0126029. doi: 10.1371/journal.pone.0126029 (PMC4416023; doi:10.1371/journal.pone.0126029)
Supplement: S3 Fig — A. Immunofluorescent images comparing XPC and UDS levels of XP21RO, T24 and HT-1197 cells to that of C5RO cells. XP21RO are known to be deficient in XPC and UDS. No difference is seen between T24 and HT-1197. Blue = DAPI, BF = Bright Field image showing C5RO cells labeled with 2 μm polystyrene beads, Green = XPC expression level, Red = UDS level. White arrows indicate the nuclei of the respective cell line. B. Immunoblot for XPC comparing HT-1197 to T24, known XPC deficient and wild type fibroblasts. C. Colony survival assay showing colony formation capacity of HT-1197 and T24 cells after UV radiation. No difference between HT-1197 and T24 is observed. Error bars indicate the standard deviation based on four independent experiments. (PDF) [file pone.0126029.s003.pdf]

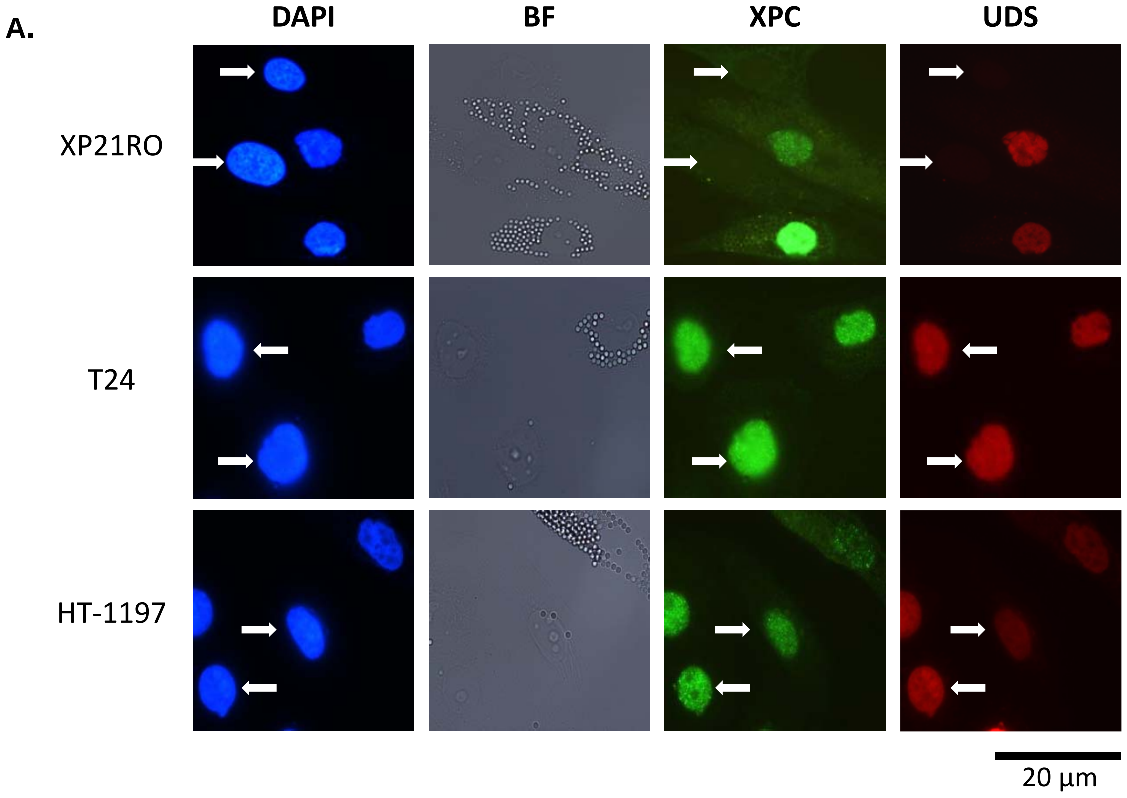


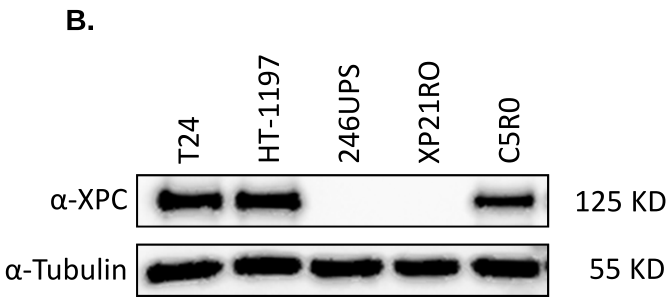


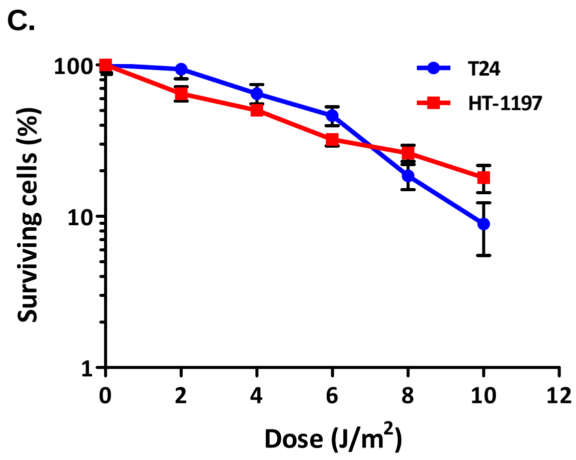


**Figure S3: HT-1197 displays normal XPC protein and UDS levels**

**A**. Immunofluorescent images comparing XPC and UDS levels of XP21RO, T24 and HT-1197 cells to that of C5RO cells. XP21RO are known to be deficient in XPC and UDS. No difference is seen between T24 and HT-1197. Blue= DAPI, BF= Bright Field image showing C5RO cells labeled with 2 µm polystyrene beads, Green= XPC expression level, Red= UDS level. White arrows indicate the nuclei of the respective cell line. **B**. Immunoblot for XPC comparing HT-1197 to T24, known XPC deficient and wild type fibroblasts. **C**. Colony survival assay showing colony formation capacity of HT-1197 and T24 cells after UV radiation. No difference between HT-1197 and T24 is observed. Error bars indicate the standard deviation based on four independent experiments.
