# Supplementary material for: Attenuated XPC Expression Is Not Associated with Impaired DNA Repair in Bladder Cancer
Source: PLoS One. 2015 Apr 30;10(4):e0126029. doi: 10.1371/journal.pone.0126029 (PMC4416023; doi:10.1371/journal.pone.0126029)
Supplement: S4 Fig — A. Tumors were split in two groups, primary tumors and recurrent tumors, and standardized XPC protein expression scores were compared from these groups. No significant difference was observed between these groups. Mann-Whitney test, p = 0.58. B. Patient follow up data indicating no difference between XPC levels from tumors that would not recur within one year of TUR and tumors that would recur. Each data point represents one tumor. Horizontal line represents median and error bars indicate interquartile range. (PDF) [file pone.0126029.s004.pdf]

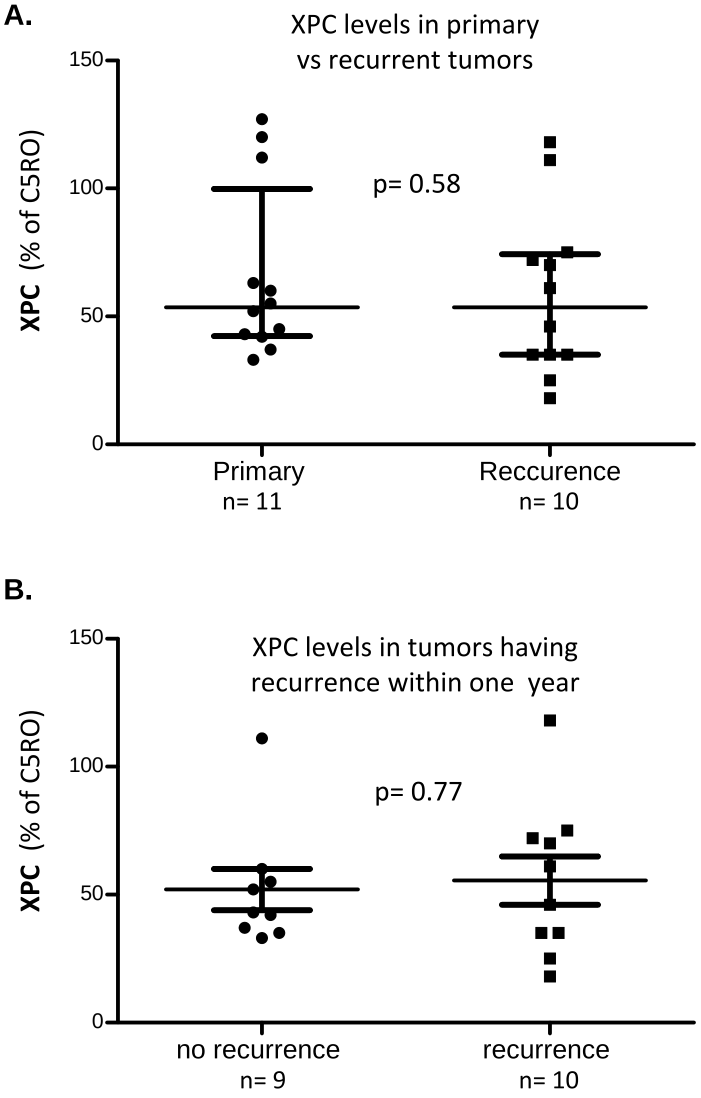

Figure S4: XPC protein levels and recurrence of tumors

A. Tumors were split in two groups, primary tumors and recurrent tumors, and standardized XPC protein expression scores were compared from these groups. No significant difference was observed between these groups. Mann-Whitney test, p=0.58 **B**. Patient follow up data indicating no difference between XPC levels from tumors that would not recur within one year of TUR and tumors that would recur. Mann-Whitney test, p=0.77. Each data point represents one tumor. Horizontal line represents median and error bars indicate interquartile range.
